# Microbial Community in a Biofilter for Removal of Low Load Nitrobenzene Waste Gas

**DOI:** 10.1371/journal.pone.0170417

**Published:** 2017-01-23

**Authors:** Jian Zhai, Zhu Wang, Peng Shi, Chao Long

**Affiliations:** 1 State Key Laboratory of Pollution Control and Resources Reuse, School of the Environment, Nanjing University, Nanjing, PR China; 2 Department of Applied Chemistry, Nanjing Polytechnic Institute, Nanjing, PR China; Natural Environment Research Council, UNITED KINGDOM

## Abstract

To improve biofilter performance, the microbial community of a biofilter must be clearly defined. In this study, the performance of a lab-scale polyurethane biofilter for treating waste gas with low loads of nitrobenzene (NB) (< 20 g m^-3^ h^-1^) was investigated when using different empty bed residence times (EBRT) (64, 55.4 and 34 s, respectively). In addition, the variations of the bacterial community in the biofilm on the longitudinal distribution of the biofilters were analysed by using Illumina MiSeq high-throughput sequencing. The results showed that NB waste gas was successfully degraded in the biofilter. High-throughput sequencing data suggested that the phylum *Actinobacteria* and genus *Rhodococcus* played important roles in the degradation of NB. The variations of the microbial community were attributed to the different intermediate degradation products of NB in each layer. The strains identified in this study were potential candidates for purifying waste gas effluents containing NB.

## Introduction

Nitrobenzene (NB) is a typical hydrophobic (Henry's law constant 2.41×10^−6^ MPa m^3^ mol^-1^ and other salient properties on the S1 Table) and refractory biodegradable volatile organic compound. NB is emitted mainly from explosives, aniline, dyes, pesticides, pharmaceutical manufacturing processes and waste water treatment plants. Because NB is relatively toxic and persistent in the environment, NB emissions are facing increasingly stringent environmental regulations. Biofilters were originally developed for odor pollution control due to their high efficiency, low energy consumption, low operating costs, and environmental-friendliness [1] and have recently been proven to be a promising technology for the treatment of volatile organic compounds (VOCs) at moderately high flow rates and low concentrations (<1000 ppm) [2–3].

Biofilters have been successfully applied to treat hydrophilic VOCs, such as styrene [4], ethanol [5], methanol [6], xylene isomers [7], hexane [8], toluene [9], phenol [10], methylamine [11], triethylamine [12], dimethylsulfide [13], and dichloromethane [14]. However, the low mass transfer of hydrophobic VOCs from the gas phase to the biofilm phase, which limits the supply of substrates to the microorganisms, results in low biodegradation rates of these compounds. Several researchers have attempted to enhance the removal of hydrophobic VOCs in biofilters by adding surfactants, applying fungal biocatalysts, and by using biofiltration with pretreatment, innovative bioreactors, and hydrophilic compounds [15].

However, to the best of our knowledge, only Oh and Bartha reported an NB-elimination capacity (EC) of 50 g m^-3^ packing h^-1^ in a trickling air biofilter [16]. In addition, few studies have investigated the relation between biofilter performance and the microbial community in NB-degrading biofilters. Microbial communities are sensitive to changes in environmental conditions; therefore, understanding the microbial ecology of biofilms in gas phase biofilters helps to optimize the design and operation of such biological treatment systems [17].

Culture-dependent conventional isolation and identification methods only provide information on relatively few microbial communities. It remains unclear whether the isolates were of any quantitative or functional significance in situ [18]. With the rapid development of analysis technologies, some advanced analysis methods, such as confocal laser scanning microscopy (CLSM), microautoradiography (MAR), polymerase chain reaction (PCR) techniques, quantitative reverse transcriptase real-time PCR (qrt-PCR), denaturing gradient gel electrophoresis (DGGE), fluorescent in situ hybridization (FISH), environmental scanning electron microscopy (ESEM), microprobe sensor techniques, laser triangulation sensor (LTC) techniques, and BIOLOG ECO-plates, have been successfully used for studying biofilms [19]. Compared with the above methods, high-throughput sequencing provides researchers with a deeper understanding of the population dynamics or functions of microorganisms in microbial communities and a new perspective for understanding the degradation processes occurring in bioreactors in their native habitat without the need of isolation [20].

In this study, gas-phase NB was treated in a biofilter, and the microbial community was analysed by high-throughput sequencing techniques. The primary objective was to clearly understand the vertical distribution of the bacterial community in a biofilter for removal of low load nitrobenzene waste gas. The present research will help enhance the degradation performance of biofilters for treating NB waste gas.

## Materials and Methods

### Biofilter setup

**Fig 1:**
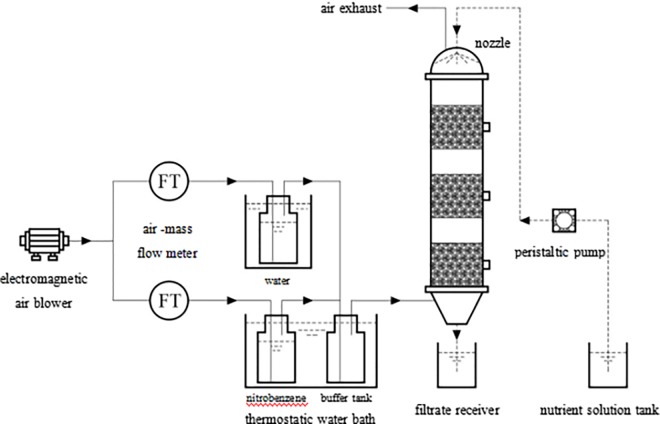
Schematic representation of the experimental setup.

A schematic of the experimental setup is illustrated in Fig 1. The biofilter reactor (inner diameter = 7 cm, effective bed height = 60 cm), which was packed with 2.3-L polyurethane foam cubes with low head loss and high porosity, was operated in a up-flow mode [21–22]. The packing parameters are summarized in Table 1. NB waste gas passed through the biofilter at a flow rate of 129.4, 149.5 and 243.5 L h^-1^, respectively. Gas sampling ports sealed with rubber septa were provided at equal intervals along the biofilter height to estimate the changes in the NB concentrations and microbial communities.

**Table 1:** The packing parameters.

parameters	value
shape	cube
size	per side 4–6 mm
bulk density	0.015 g/cm^3^
water holding capacity	55 g-H_2_O/g
porosity	95%
average pore size	0.8 mm
specific surface area	73.65 m^2^/g

Values provided by the manufacturer.

The biofilter was inoculated using aerobic activated sludge from the secondary sedimentation tank of an NB production waste water treatment plant (WWTP). The mixed culture was re-circulated through the packed bed bioreactor using a peristaltic pump (WT600-1F, Baoding longer Pump Ltd., CHN) at a rate of 0.5 L min^-1^ until a visible biomass was noticed on the support material surface. The moisture content of the bed was maintained at 75%-85% by periodically adding (once a day) fresh mineral salt media. The trickling density was maintained at 0.52 m^3^ m^-2^ h^-1^ throughout the experiments. The mineral salt nutrient solution contained the following components (per litre in distilled water): 0.5 g K_2_HPO_4_, 0.1 g MgSO_4_·7H_2_O, 4.5 g KH_2_PO_4_, 2 g NH_4_Cl, and 2 mL of vitamins and trace minerals [23]. The original pH of the nutrient solution was adjusted to 7.0.

### Analytical methods

The NB concentrations in the gas samples were measured by gas chromatography (GC) (2014, SHIMADZU, USA) using a Rtx-5 column (30 m, 0.32 mmID, 0.5 μm) and an FID. The flow rates were 30 mL min^−1^ for H_2_ and 300 mL min^−1^ for air. Nitrogen was used as the carrier gas at a flow rate of 2 mL min^−1^. The temperatures located at the GC injection, oven and detection ports were 150°C, 220°C and 250°C, respectively. The pH value of the filter liquor was measured by a pH metre (FEP20, Mettler-Toledo, CHE). The nitrite concentration was monitored according to the Chinese National Standard GB 7493–87 water quality-determination of nitrogen (nitrite)- spectrophotometric method [24].

### Abiotic experiment

The NB abiotic experiment was performed with a biofilter containing a clean packing medium (2.3 L) to determine the amount of NB removed by abiotic means.

### Sample collection, DNA extraction and polymerase chain reaction (PCR)

The representative biological samples were collected from the sampling port within the biofilter at the end of the experiment (EBRT 55.4 s). The performance of the biofilter was stable during this stage (ILR < 20 g m^-3^ h^-1^ in accordance with engineering practice). Each sample was immersed in 20 mL of sterilized water and sonicated for 20 s to strip the biomass from the packing material. At room temperature, 2 mL samples were then centrifuged at 13,000 rpm for 2 min using an Eppendorf centrifuge 5417R (Eppendorf, Hamburg, GER). The DNA template was extracted using a FastDNA^®^ Spin Kit for Soil (MP Biomedicals, CA, USA) according to the manufacturer’s instructions.

DNA samples (10 mg each) were employed to generate amplicons that covered the V3-V5 hypervariable regions of the bacterial 16S rRNA. PCRs were performed in 20 μL reaction mixtures that each contained 1 μL of the DNA template with approximately 10 ng of DNA, 0.8 μL (5 μM) of the forward (338 F(5'- ACT CCT ACG GGA GGC AGC A-3')) and reverse (806 R(5'- GGA CTA CHV GGG TWT CTA AT-3')) primers, 2 μL (2.5 mM) of deoxynucleoside triphosphate (dNTPs), 0.4 μL of Fast Pfu Polymerase, 4 μL of 5×FastPfu Buffer and complement ddH_2_O. The DNA was amplified by using a GeneAmp® (9700, ABI, USA). PCR amplification was performed using initial denaturation at 95°C for 3 min, followed by 27 cycles of denaturation at 95°C for 30 s, annealing at 55°C for 30 s, extension at 72°C for 45 s and a final extension at 72°C for 10 min. Amplicons were extracted from 2% agarose gels and purified using an AxyPrep DNA Gel Extraction Kit (Axygen Biosciences, Union City, CA, USA) according to the manufacturer’s instructions and were quantified using QuantiFluor™ -ST (Promega, USA).

### Illumina MiSeq high-throughput sequencing

Purified amplicons were pooled in equimolar and paired-end sequences (2×250) on an Illumina MiSeq platform (PE250, Illumina, USA) according to the standard protocols. Corresponding sequences with specific barcodes inside each sample were selected using Mothur (http://www.mothur.org/). Then, data denoising was conducted as described in a previous study [25]. After noise reduction, 23,600 reads were drawn randomly from the samples to compare all the samples at the same sequencing depth. Downstream taxonomic assignment was performed using the Ribosomal Database Project (RDP) (http://rdp.cme.msu.edu/) classifier a with confidence threshold of 50%. Mothur was applied to calculate the richness and diversity indices, including the operational taxonomic units (OTUs), Chaos index, and Shannon index [26]. A cluster analysis of the microbial community was conducted using PAleontological STatistics (PAST, v.3.01) software with an unweighted pair-group average method.

### Calculations

The empty bed residence time (EBRT, s), removal efficiency of NB (RE, %), inlet loading rate of NB (ILR, g m^–3^ h^–1^) and the elimination capacity of NB (EC, g m^–3^ h^–1^) of the reactor were estimated by using the following equations:

Emptybedresidencetime,EBRT=V/Qs (1)

$$Removal\;efficiency,\;RE=\left(C_{i}\text{-}C_{o}\right)\;/\;C_{i}\;*\;100\%\;\%$$ (2)

$$Inlet\;loading\;rate,\;ILR=QC_{i}/\;V\;g\;m^{\text{-}3}\;h^{\text{-}1}$$ (3)

$$Elimination\;capacity,\;EC=Q\left(C_{i}\text{-}C_{o}\right)\;/\;V\;g\;m^{\text{-}3}\;h^{\text{-}1}$$ (4)

where V is the volume (m^3^) of the packed bed section, Q is the gas flow rate (m^3^ h^-1^), and C_i_ and C_o_ are the inlet and outlet concentrations (mg m^-3^) of NB, respectively.

## Results and Discussion

### NB removal by abiotic means

During the abiotic experiment, 100 mg m^-3^ gas-phase NB was introduced to the biofilter at a flow rate of 149.5 L h^-1^ for a period of 7 days, and the NB removal efficiency was measured under the trickling density of a mineral salt nutrient solution of 0.52 m^3^ m^-2^ h^-1^. The packing medium removed less than 2.47% of the inlet NB from the gas stream (data not shown), which showed that the effect of the NB abiotic degradation was negligible in the biofilter.

### NB removal by the biofilter

**Fig 2:**
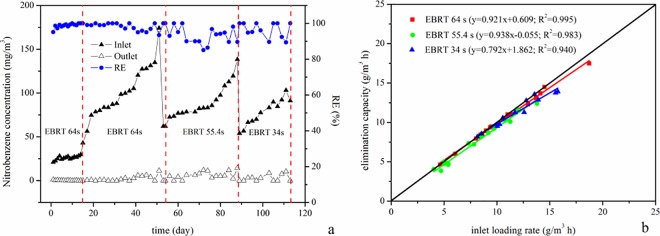
Performance of the biofilters for the removal of NB: effect of the EBRT (a) and the elimination capacity vs. the inlet loading rate (b).

**Fig 3:**
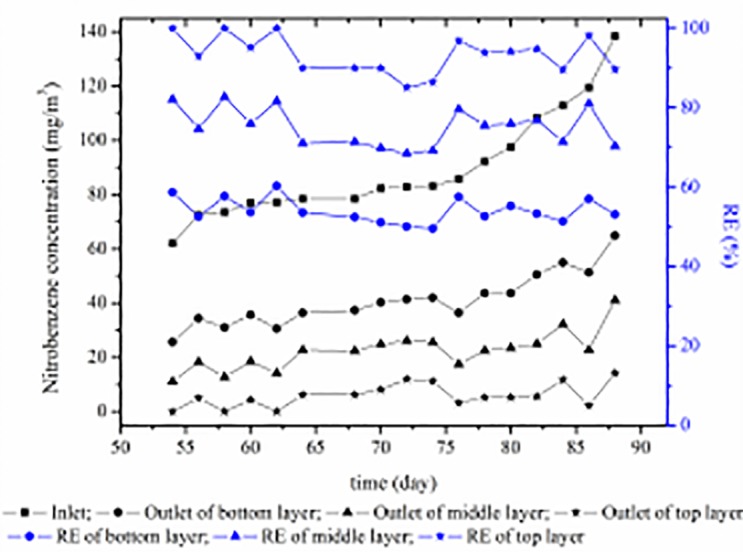
Outlet concentrations of NB and REs in different layers with an EBRT of 55.4 s.

Generally, most laboratory-scale biofilters were fed with only low concentrations of pollutants during acclimation. In this study, 20–30 mg m^-3^ gas-phase NB was introduced to the biofilter with an empty bed residence time (EBRT) of 64 s. The biofilter achieved a nearly stable RE value of 98.8% after 14 days (Fig 2A), which indicated that the biofilter inoculated with aerobic activated sludge from the NB waste water treatment plant required a shorter acclimation time. After the acclimation period, steady-state experiments were carried out at different EBRTs and inlet NB concentrations, which are important parameters that affect the efficiency of biofiltration units. As depicted in Fig 2A, when the inlet concentrations of NB gradually increased from 43.0 to 174.2 mg m^-3^ with an EBRT of 64 s during days 15–53, the RE values of NB fluctuated between 93.5% and 100%. The EBRT decreased from 64 s to 55.4 s during the next period from the 54th day to the 88th day (Fig 3), the inlet concentrations of NB were maintained in the range of 62.0–138.4 mg m^-3^ and the outlet concentrations of NB in the bottom layer, middle layer and top layer were 25.6–64.9 mg m^-3^, 11.1–41.1 mg m^-3^, and 0–14.3 mg m^-3^, respectively. RE values of 49.5%-60.3%, 68.5%-82.7%, and 85.1–100% were achieved in the bottom layer, middle layer and top layer, respectively. Finally, the biofilter was subjected to a much lower EBRT (34 s) and RE values of 89.4%-100% were maintained during days 89–113 (Fig 2A). These data indicate that the biofilter performed well and that the RE was hardly relevant to the EBRT with low NB concentrations.

In this study, maximum EC values of 17.5, 12.4 and 13.9 g m^-3^ h^-1^ were achieved with EBRT values of 64, 55.4 and 34 s and ILR values of 18.7, 13.8 and 15.7 g m^-3^ h^-1^, respectively (Fig 2B). The slope of the linear fitting equation of NB degradation with an EBRT of 64 s (0.921) was higher than that with an EBRT of 34 s (0.792) and was similar to that with an EBRT of 55.4s (0.938), which was near 1.0 under low NB loads. In this region, the EC of the biofilter is highly dependent on the EBRT. Increasing the given EBRT enhanced the transfer rate of NB from the gas-phase to the biofilm and improved the EC [27].

### Overall microbial community diversity

**Fig 4:**
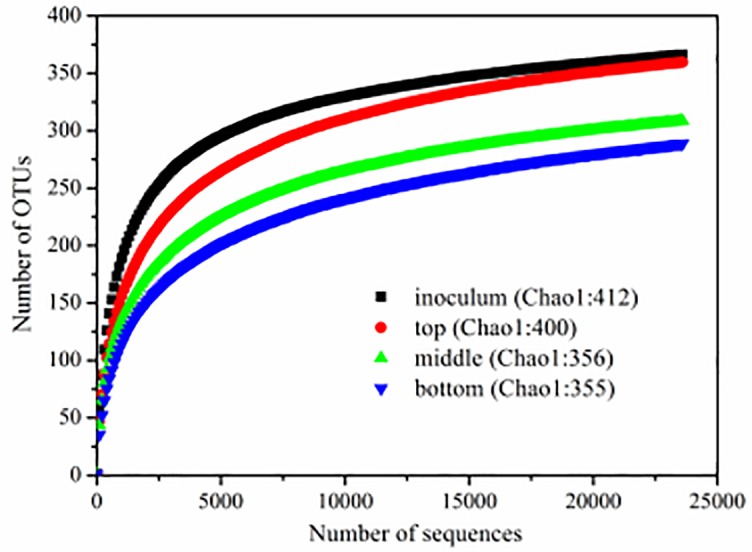
Rarefaction curves based on 16S rRNA gene sequencing of the samples (from top, middle and bottom layers) and the initial inoculum. The Chao1 index is also shown. The OTUs were defined by a 97% cutoff.

Samples were collected from the initial inoculum in the top, middle and bottom layers of the biofilter with an EBRT of 55.4 s, respectively. The microbial community diversities and phylogenetic structures of these samples were analysed by using Illumina MiSeq high-throughput sequencing. Over 20000 sequences were obtained for each sample. The Good’s coverages of these samples ranged from 99.76% to 99.89%, indicating that the sequences generated at this sequencing depth represented the bacterial communities of these samples well [26]. For fair comparison, the library size of each sample was normalized to the same sequencing depth (23,600 reads) by randomly removing the redundant reads. Relatively higher OTUs were found in the initial inoculum (366) than the samples of the top (360), middle (309) and bottom layers (289). The estimated species richness Chao1 for each sample was also consistent with this trend (Fig 4). Based on the Shannon index, the initial inoculum (4.60) also had a relatively higher diversity than the sample of the top layer (4.12), followed by the samples of the middle (3.84) and bottom layer (3.43). The biodiversity variation trends were attributed to the NB waste gas acting as the sole carbon and energy source to the biofilter, which decreased the biodiversity of each layer in the biofilter. During the continuous biodegradation of NB, the increment of intermediate product types may have resulted in the increment of biodiversity from the bottom layer to the top layer in the biofilter.

### Shifts in microbial community structure and composition

**Fig 5:**
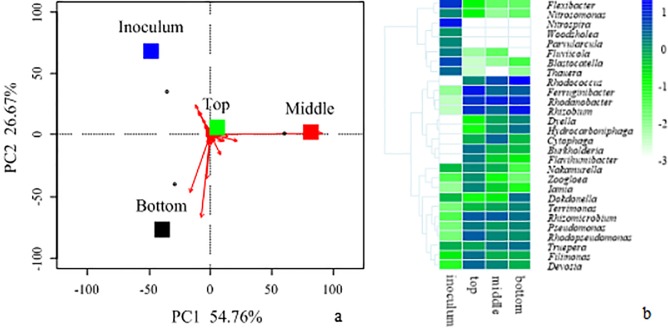
**Principal component analysis (PCA) of bacterial communities from the initial inoculum and samples of the top, middle and bottom layers based on the classified OTUs (a)**. **Heat map illustrating the abundances of all the major genera (with a relative abundance of more than 1% in at least one sample).** The color intensity (log scale) in each panel indicates the relative abundance of the genus in each sample (b).

**Fig 6:**
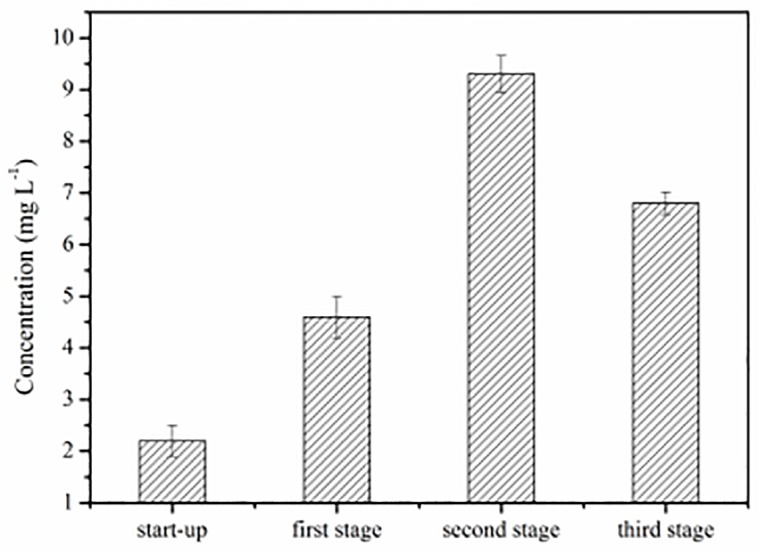
Concentrations of nitrite released.

The small differences between the microbial community structures in the three layers of the biofilter were distinguished by using principal component analysis (PCA) and from the abundance of major genera (Fig 5). PCA revealed that the bacterial community structure varied significantly among the layers of the biofilter. Principle component 1 (PC1) and principle component 2 (PC2) accounted for 54.76% and 26.67% of the total variance, respectively. As demonstrated by PCA (Fig 5A), the initial inoculum was located in the first quadrant, samples of the top and middle layer were located in the second quadrant, and samples of the bottom layer were located in the third quadrant. Interestingly, the samples of the top layer were not near the samples of the middle and bottom layers or the initial inoculum. The heat map shows that the major genera of each layer were different from each other (Fig 5B). In this study, nitrite was detected in the filter liquor (Fig 6). In addition, nitrite and catechol were characterized as the metabolic products of NB oxidation under aerobic conditions [28]. This result suggests that the subtle variations of the bacterial community structure are attributed to the accumulation of NB metabolites.

**Fig 7:**
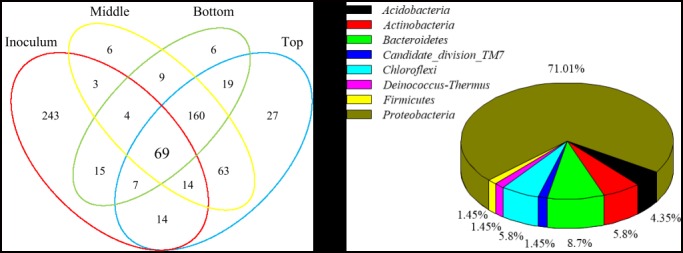
Venn diagram illustrating the overlap of the four bacterial communities from the initial inoculum and the samples of the top, middle and bottom layers. The shared OTUs (69) were analysed at the phylum level.

The total classified number of OTUs in all four bacterial communities was 659, and 69 OTUs (10.47%) of the OTUs were shared by the four communities. More than 71.01% of the shared OTUs belonged to *Proteobacteria* according to the phylum-level taxonomic distribution of the four samples (inoculum and top, middle and bottom layers), and the OTUs belonging to *Bacteroidetes* (8.70%), *Actinobacteria* (5.80%), *Chloroflexi* (5.80%) and *Acidobacteria* (4.35%) were shared at similar levels (Fig 7). The data showed that the microbes in the phylum *Proteobacteria* were key during the removal process of NB from waste gas with low amounts of NB.

**Fig 8:**
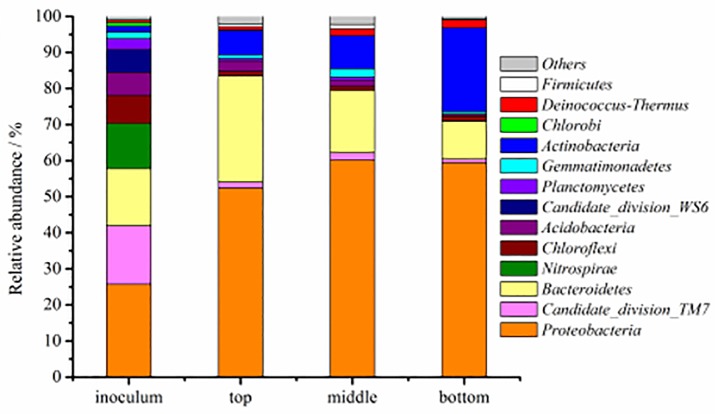
Abundances and distributions of the different phyla in the four biofilm samples.

A total of 14 phyla were identified, among which 14, 12, 12 and 12 were detected from the initial inoculum and the samples of the top, middle and bottom layers, respectively. *Proteobacteria* were a dominant part of the bacterial community in the initial inoculum (25.73%), top layer (52.36%), middle layer (60.16%) and bottom layer (59.45%), respectively. The relative abundance of *Bacteroidetes* was 29.52% in the top layer, which was higher than those in the middle layer (17.23%), bottom layer (10.46%) and the initial inoculum (15.86%). The relative abundance of *Actinobacteria* decreased from 23.24% (bottom layer) to 9.18% (middle layer), 6.69% (top layer) and 1.63% (the initial inoculum) (Fig 8). From Fig 3, NB was removed mainly in the bottom layer (49.5%-60.3%) and middle layer (68.5%-82.7%); therefore, it can be concluded that the phylum *Actinobacteria* and *Proteobacteria* had major contributions to the degradation of NB, and the main function of the phylum *Bacteroidetes* was to degrade the NB metabolic intermediates.

### Potential functions and relative abundances of the dominant genera

A total of 89 genera were classified among the test samples. Twelve genera with a relative abundance >1% in the initial inoculum and samples of the top, middle and bottom layer communities are shown in Table 2. *Nitrospira* (12.56%), *Flexibacter* (7.08%) and *Blastocatella* (5.05%) were obviously dominant in the initial inoculum.

**Table 2:** Genera (relative abundance>1% at genus levels) in the initial inoculum and the samples of the top, middle and bottom layers. The dominant genera in the different layers are shown in bold.

Phylum	Class	Genus (%)	Initial	Top	Middle	Bottom
*Nitrospirae*	*Nitrospira*	*Nitrospira*	12.56	0.00	0.00	0.00
*Bacteroidetes*	*Sphingobacteria*	*Flexibacter*	7.08	0.17	0.02	0.02
*Proteobacteria*	*α-proteobacteria*	*Blastocatella*	5.05	0.00	0.01	0.04
*Deinococcus-Thermus*	*Deinococci*	*Truepera*	0.65	0.83	1.87	2.16
*Proteobacteria*	*α-proteobacteria*	*Devosia*	0.08	2.59	1.46	0.72
*Proteobacteria*	*α-proteobacteria*	*Rhizomicrobium*	0.04	3.09	3.22	2.32
*Proteobacteria*	*α-proteobacteria*	*Rhodopseudomonas*	0.02	3.62	1.35	1.21
*Proteobacteria*	***γ-****proteobacteria*	***Pseudomonas***	0.02	1.92	0.93	1.58
*Proteobacteria*	*α-proteobacteria*	***Ferruginibacter***	0.01	14.74	2.98	3.22
*Proteobacteria*	***γ-****proteobacteria*	***Rhodanobacter***	0.00	7.70	10.35	8.36
*Proteobacteria*	*α-proteobacteria*	*Rhizobium*	0.00	8.01	2.45	12.86
*Actinobacteria*	*Actinobacteria*	***Rhodococcus***	0.00	1.43	4.48	22.18

In the bottom layer, genus *Rhodococcus* (22.18%), *Rhizobium* (12.86%) and *Rhodanobacter* (8.36%) had obviously higher relative abundance than in the initial inoculum (Table 2). The genus *Rhodococcus* belonged to the phylum *Actinobacteria*, which is known to be capable of NB biodegradation [29–31]. According to Fig 3, the RE of NB was 49.5%-60.3% in the bottom layer; thus, it was concluded that the genus *Rhodococcus* was the major NB-degrading genus. This finding may explain why *Rhodococcus* was dominant in the inlet of the biofilter. In the middle layer, the genus *Rhodanobacter* was obviously enriched (10.35%); however, the relative abundance of *Rhodococcus* in the initial inoculum was low (0.004%). *Rhodococcus* belongs to the phylum *Proteobacteria*, which is capable of denitrification [32–33]. Nitrite is a type of metabolite that is used for NB oxidation, which explains the predominance of *Rhodanobacter* in this layer [34]. In the top layer, the genera *Ferruginibacter* (14.74%), *Rhizobium* (8.01%) and *Rhodanobacter* (7.70%) exhibited exceedingly higher abundance than those in the initial inoculum. The genus *Ferruginibacter* belongs to the phylum *Bacteroidetes*, which has frequently been detected at wastewater treatment plants [35–36]. A previous study indicated that members of *Ferruginibacter* are capable of hydrolysing some organic matter [37]. It was asserted that the genus *Ferruginibacter* predominated in the top layer because of the degradation of the NB metabolic intermediates. Although *Pseudomonas* possessed a fairly lower abundance in the initial inoculum (0.02%) and top (1.92%), middle (0.93%) and bottom (1.58%) layers, respectively, *Pseudomonas* has also been reported to have the ability to degrade high concentrations of NB [38].

## Conclusions

This study demonstrated an efficient system for NB removal at low ILR values. The REs were always higher than 93% at different phases, and the slopes of EC/ILR were close to 1.0. Illumina MiSeq high-throughput sequencing technology was successfully applied to analyse the variations of the biodiversity and microbial community structure in these biofilms along the longitudinal direction of the biofilter. The increase of biodiversity from the bottom up was attributed to the increase of NB metabolic intermediate types. The microbial community structure affected the ecological function significantly and further influenced the performance of the biofilter. The sequencing data showed that the phylum *Actinobacteria* and *g*enus *Rhodococcus* played key roles in the degradation of NB in the bottom layer.

## Supporting Information

S1 Table Nitrobenzene properties. (DOCX)
