# Supplementary material for: Microbial Community in a Biofilter for Removal of Low Load Nitrobenzene Waste Gas
Source: PLoS One. 2017 Jan 23;12(1):e0170417. doi: 10.1371/journal.pone.0170417 (PMC5256912; doi:10.1371/journal.pone.0170417)
Supplement: S1 Table — (DOCX) [file pone.0170417.s001.docx]

**S1 Table**. Nitrobenzene properties

| Properties | |
| --- | --- |
| Chemical formula | C_6_H_5_NO_2_ |
| Molar mass | 123.06 g mol^-1^ |
| Density | 1.199 g cm^-3^ |
| Appearance | yellowish, oily liquid |
| Odor | pungent, like paste shoe polish |
| Melting point | 5.7 °C (42.3 °F; 278.8 K) |
| Boiling point | 210.9 °C (411.6 °F; 484.0 K) |
| Henry's law constant | 2.41×10^-6^ MPa m^3^ mol^-1^ |
| Solubility in water | 0.19 g 100 mL^-1^ at 20 °C |
| Vapor pressure | 0.3 mmHg (25°C) |
